# Cell-free culture supernatants of *Lactobacillus* spp. and *Pediococcus* spp. inhibit growth of pathogenic *Escherichia coli* isolated from pigs in Thailand

**DOI:** 10.1186/s12917-022-03140-8

**Published:** 2022-01-29

**Authors:** Thotsapol Kaewchomphunuch, Thunyathorn Charoenpichitnunt, Varissara Thongbaiyai, Natharin Ngamwongsatit, Kampon Kaeoket

**Affiliations:** Department of Clinical Sciences and Public Health, Faculty of Veterinary Science, Mahidol University, 999 Phuttamonthon 4 Rd., Salaya, Phuttamonthon, Nakhon Pathom, 73170 Thailand

**Keywords:** Antimicrobial peptides, *Escherichia coli*, Inhibition, *Lactobacillus* spp., *Pediococcus* spp., Pigs

## Abstract

**Background:**

Pathogenic *Escherichia coli* (*E. coli*) is an important causative agent for infectious diseases in pigs and causes significant economic loss. The global concern of antimicrobial resistance of bacteria raises awareness of the alternative ways of using antimicrobial peptides (AMPs). The study was aimed to identify and test the efficacy of AMPs from *Lactobacillus* spp. against the growth of pathogenic *E. coli* isolated from pigs in Thailand. Briefly, cell-free culture supernatants (CFCS) from 3 strains of lactic acid bacteria (LAB) consisting of *Lactobacillus acidophilus* (strain KMP), *Lactobacillus plantarum* (strain KMP), and *Pediococcus pentosaceus* (strain KMP) were tested against pathogenic *E. coli* via agar well diffusion assay in quadruplicates. The presence of a zone of inhibition (ZOI) around wells was evaluated at different incubation time. Acid and bile tolerance test was performed for bacterial viability in acid and bile salt conditions. In addition, LAB cross-streaking assay was evaluated for antagonist activity.

**Results:**

The study showed that CFCS from *L. acidophilus* KMP, *L. plantarum* KMP, and *P. pentosaceus* KMP could inhibit the growth of pathogenic *E. coli* isolated from pigs in a time-dependent manner. To exemplify, the ZOI of *L. plantarum* KMP against *E. coli* (ETEC) at 8, 10, 12, 14, and 16 h incubation, were 26.6 ± 1.1, 24.9 ± 1.9, 22.5 ± 2.4, 20.3 ± 2.9, and 17.9 ± 3.3 mm, respectively. The ZOI was significantly different between 8, 10, 12, 14 h incubation, and the ZOI of the CFCS from *L. plantarum* KMP was larger than others (*P*-value < 0.05). Furthermore, *L. acidophilus* KMP, *L. plantarum* KMP, and *P. pentosaceus* KMP showed viability in pH 3.0, 0.3, and 0.5% (w/v) bile salt concentration. They exhibited no antagonist activity among each other.

**Conclusions:**

According to the results, the CFCS from LAB including *L. acidophilus* KMP, *L. plantarum* KMP and *P. pentosaceus* KMP can inhibit the growth of pathogenic *E. coli*, isolated from pigs in Thailand. The antimicrobial activity observed was incubation time dependent.

**Supplementary Information:**

The online version contains supplementary material available at 10.1186/s12917-022-03140-8.

## Background

*Escherichia coli* (*E. coli*) is classified in family of *Enterobacteriaceae*. Feature characteristics are Gram-negative, rod-shaped, non-spore forming, flagellated, facultative anaerobic, and glucose fermentation bacteria [1, 2]. *E. coli* is an important causal agent of infectious diseases in pigs. There causes pathogenicity in wide range of systemic [1]. In particular, swine enteric colibacillosis associated with 2 main pathotypes, enterotoxigenic *E. coli* (ETEC) and enteropathogenic *E. coli* (EPEC) [3, 4]. The ETEC colonizes at intestinal epithelium and produces toxin inducing electrolyte imbalance and fluid homeostasis disturbance [5, 6]. As a result, there cause watery diarrhea and edema disease in neonatal and post-weaning pigs [1, 7]. These impact on significantly economic losses in pig industry due to high mortality and morbidity rate as well in additional costs for prophylaxis and treatment [3]. The economic loss in post-weaning diarrhea (PWD) is estimated as a financial costs in range from €2 to €6.07 per piglet [8]. The preventive strategies for prevention and control swine colibacillosis are to maintain biosecurity system, decrease quantities of pathogenic *E. coli* in environment, maintain neonatal high level of immunity, and *E. coli* strains vaccination [3].

Antimicrobial drugs, such as β-lactam, cephalosporins, aminoglycosides, polymyxins, sulphonamide combined with trimethoprim, and fluoroquinolones are required in many cases of the disease on a farm [3], but the usage of antimicrobial drugs without the supervision of veterinarians can lead to the emergence of resistant bacteria and the limitation of drug alternatives to treat the diseases. Recently, antimicrobial resistance genes, such as *mcr-1* gene encoding for polymyxin resistance, were identified in drug-resistant bacteria, including *E. coli*. In addition, the resistance can be transferred from livestock to other animals, humans, and the environment [9–11].

The increasing awareness about antimicrobial drug resistance leads to the need to study alternative ways to prevent infections in pigs to reduce economic losses in pig farms. Some studies show non-antibiotic feed additives can improve immune response in pig intestine and create the proper environment for normal flora in the gastrointestinal tract of pig [12]. Those feed additives consist of acidifiers, zinc and copper, prebiotics, yeast products, and probiotics such as *Lactobacillus* spp. [13]. Antimicrobial peptides (AMPs) are also widely used as alternative antibiotics [14, 15].

AMPs are small oligopeptides with 12–50 amino acids with amphipathic structure consisting of hydrophilic regions, hydrophobic regions, and cation. There are many criteria to categorize AMPs. Classification is based on sources, activities, amino acid-rich species, and structure-based characteristics [16]. AMPs have broad-spectrum activity against bacteria, fungi, eukaryotic parasites, and viruses [16, 17]. In antibacterial activity, the cationic character of AMPs plays an essential role in strong interaction with anionic character of phospholipid head groups (i.e., cardiolipin and phosphatidylglycerol) in bacterial cell membrane [18, 19]. This mechanism of action induces membrane disruption and leads pore formation and intracellular substance leakage then the bacterial cell lysis and death [20]. The other mechanism of action is described with intracellular inhibitory activities, a biosynthesis and metabolism inhibition of nucleic acid and protein [21].

Lactic Acid Bacteria (LAB) are group of bacteria in genera of *Lactobacillus*, *Pediococcus*, *Enterococcus*, *Streptococcus*, *Lactococcus*, and *Leuconostoc* species [22, 23]. Their feature characteristic is to produce antimicrobial substances to inhibit other pathogens. The antimicrobial substances can be AMPs (bacteriocins), organic acids (lactic acid), organic compound (diacetyl), and acidic gas (carbon dioxide). According to the antimicrobial properties, they provide benefits in food bio-preservation of food safety, probiotics dietary supplement and livestock production as well [22].

Cell-free culture supernatants (CFCS) produced from LAB (LAB-CFCS) contain active substances and bacteriocins [24]. There have been demonstrated that LAB-CFCS can inhibit the growth of pathogenic bacteria *E. coli* O157:H7 [25], *Gardnerella vaginalis* [26], *Listeria monocytogenes* [27], *Salmonella* Typhi, *Salmonella *Typhimurium [28], *Shigella flexneri* [29, 30]*, Shigella sonnei* [30], *Staphylococcus aureus* [31], and *Streptococcus suis* [32]. However, the studies of the efficacy of LAB-CFCS with its AMPs against pathogenic *E. coli* in pigs are scarce and under investigation [33]. Hence, this paper aims to test the efficacy of CFCS from *Lactobacillus* spp. and *Pediococcus* spp. against the growth of pathogenic *E. coli* isolated from pigs.

## Results

### Agar well diffusion assay

**Fig. 1:**
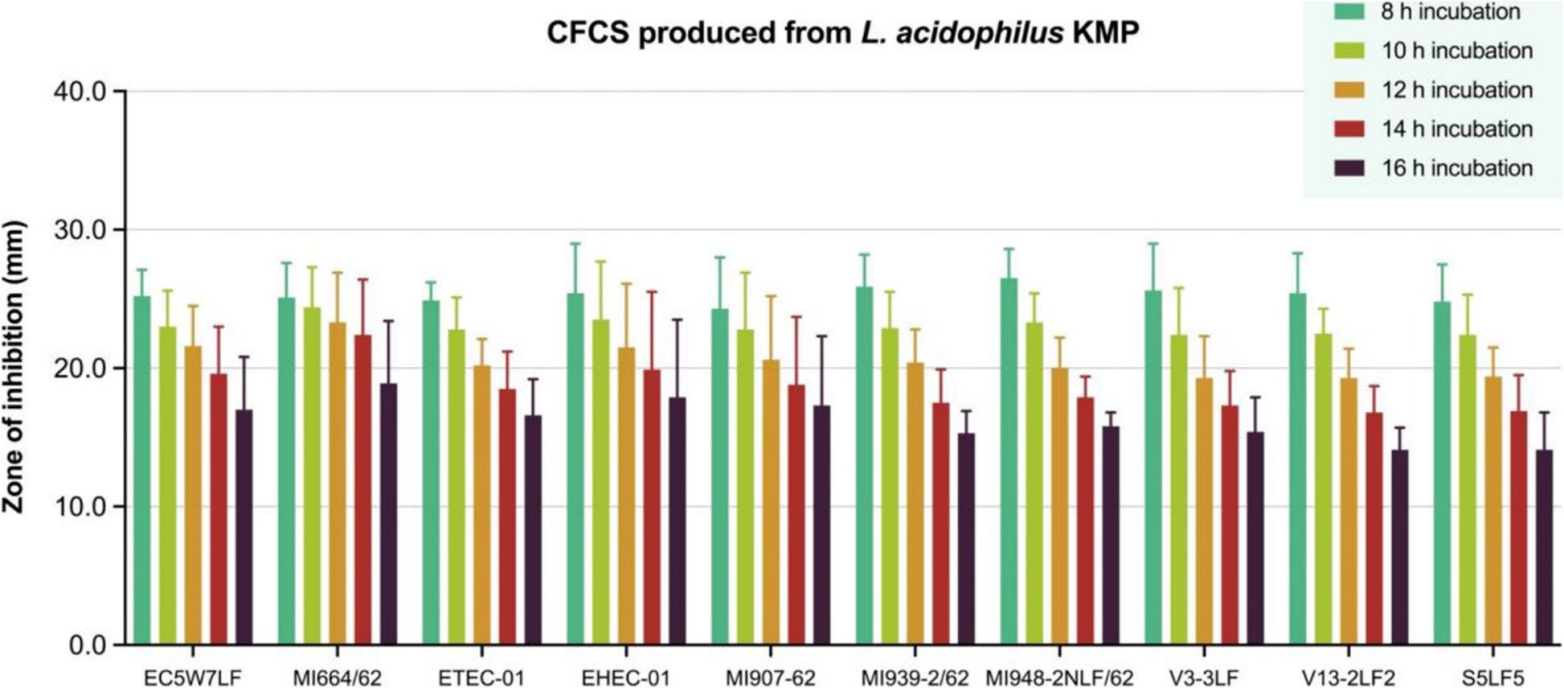
The antibacterial activities in different hours of incubation of cell-free culture supernatant (CFCS) of *L. acidophilus* KMP against 10 strains of pathogenic *Escherichia coli* in agar well diffusion assay, expressed as mean with standard deviation (*n* = 4)

**Fig. 2:**
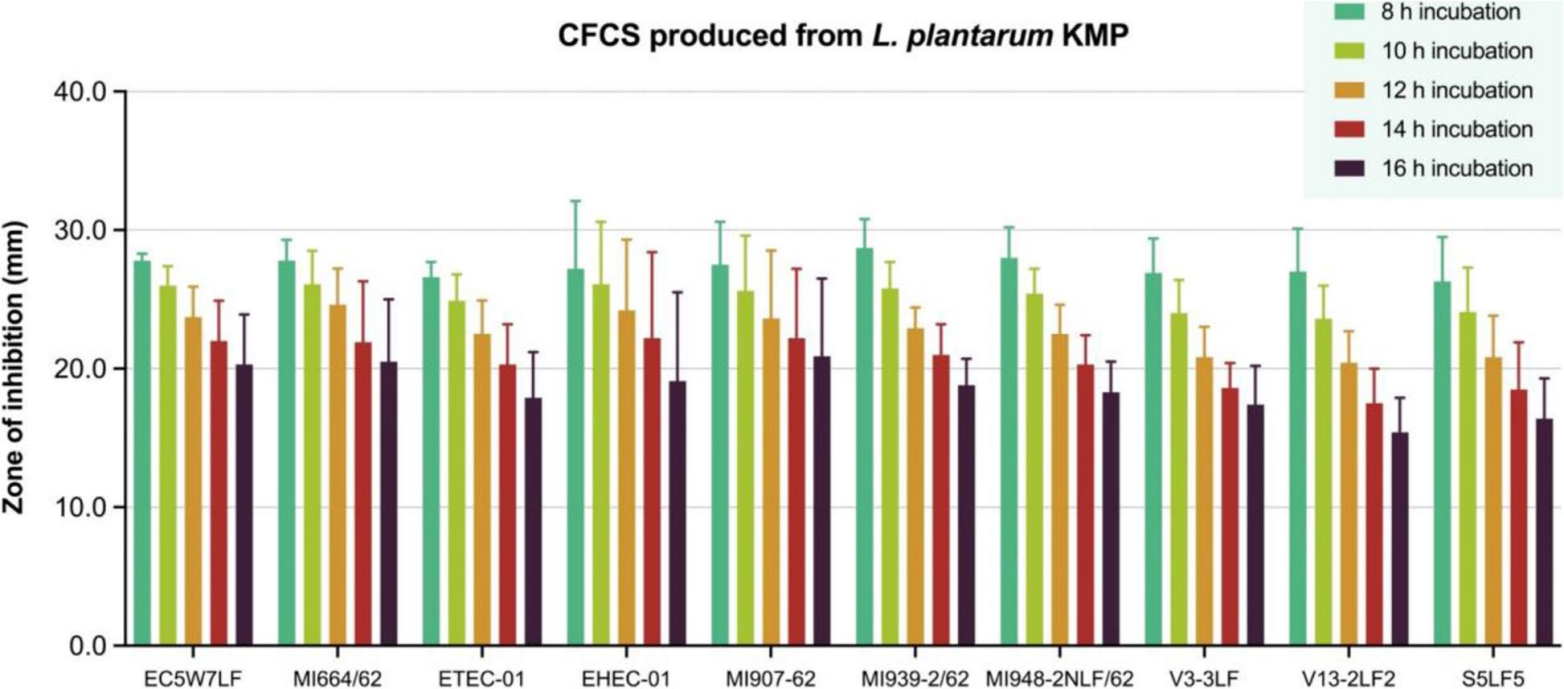
The antibacterial activities in different hours of incubation of cell-free culture supernatant (CFCS) of *L. plantarum* KMP against 10 strains of pathogenic *Escherichia coli* in agar well diffusion assay, expressed as mean with standard deviation (*n* = 4)

**Fig. 3:**
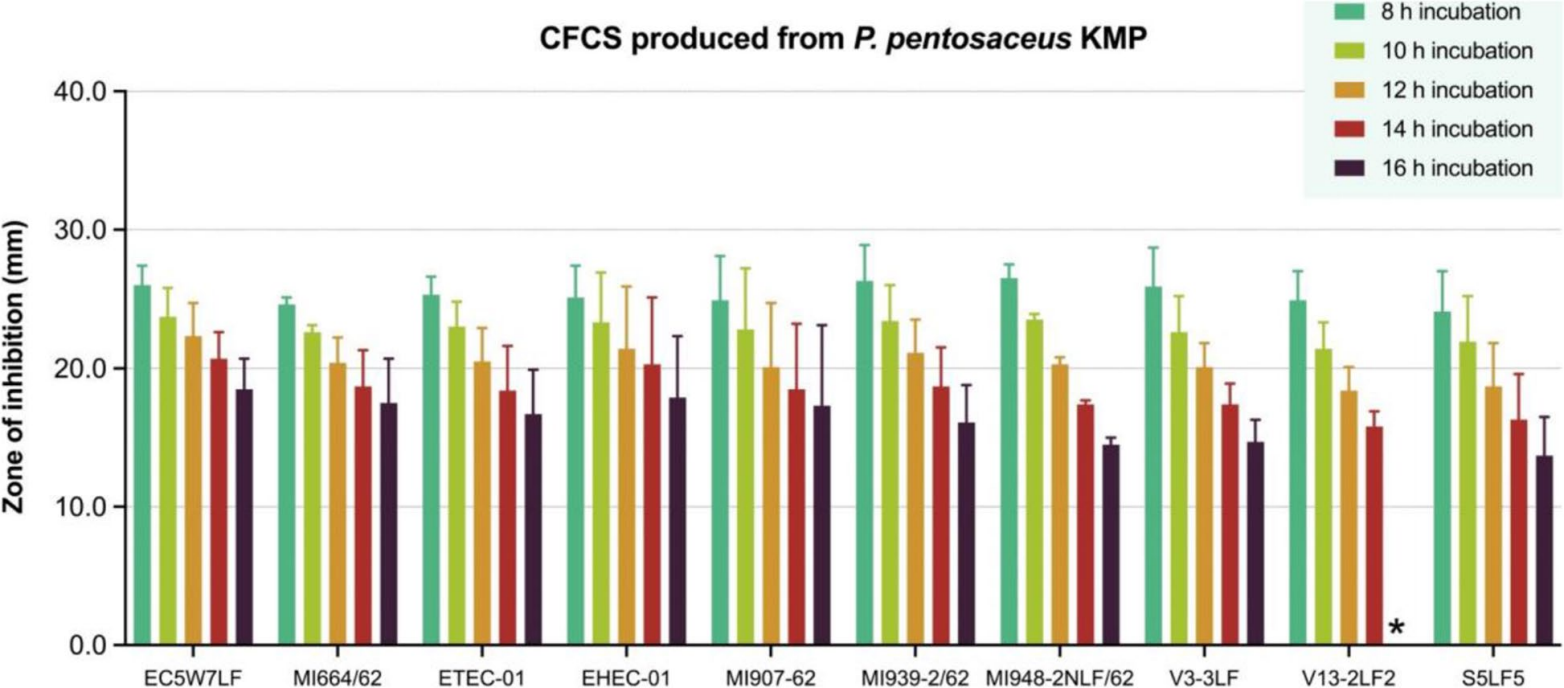
The antibacterial activities in different hours of incubation of cell-free culture supernatant (CFCS) of *P. pentosaceus* KMP against 10 strains of pathogenic *Escherichia coli* in agar well diffusion assay, expressed as mean with standard deviation (*n* = 4)

To evaluate inhibitory activities of LAB-CFCS produced from *L. acidophilus* KMP, *L. plantarum* KMP and *P. pentosaceus* KMP, the LAB-CFCS was performed by agar well diffusion assay. Zone of inhibition (ZOI) was observed at 8, 10, 12, 14, and 16 h incubation time. The results of inhibitory activities were shown in Figs. 1, 2, 3. A negative control (MRSC broth) showed absence of ZOI in all of experiments.

The ZOI of CFCS from *L. acidophilus* KMP against all 10 strains of pathogenic *E. coli* were presented in Fig. 1 and more details (see Additional file 1). At 8 h incubation, the ZOI varied from 24.3 to 26.5 mm. At 16 h incubation, the range of ZOI was significantly decreased in from 14.1 to 18.9 mm. However, all ZOI were absent at 18 h incubation.

The ZOI of CFCS from *L. plantarum* KMP against all 10 strains of pathogenic *E. coli* were presented in Fig. 2 and more details (see Additional file 1)*.* At 8 h incubation, the range of ZOI was between 26.3 to 28.7 mm. At 16 h incubation, the range of ZOI was significantly decreased in between 15.4 to 20.9 mm. However, all ZOI were absent at 18 h incubation.

The ZOI of CFCS from *P. pentosaceus* KMP against all 10 strains of pathogenic *E. coli* were presented in Fig. 3 and more details (see Additional file 1). At 8 h incubation, the range of ZOI was between 24.1 to 26.5 mm. At 16 h incubation, the range of ZOI was significantly decreased in between 0.0 to 18.5 mm. In addition, the inhibitory activity against *E. coli* V13-2LF2 had been terminated at 14 h incubation with the presence of a ZOI of 15.8 mm. However, in other strains the ZOIs were absent at 18 h incubation.

**Fig. 4:**
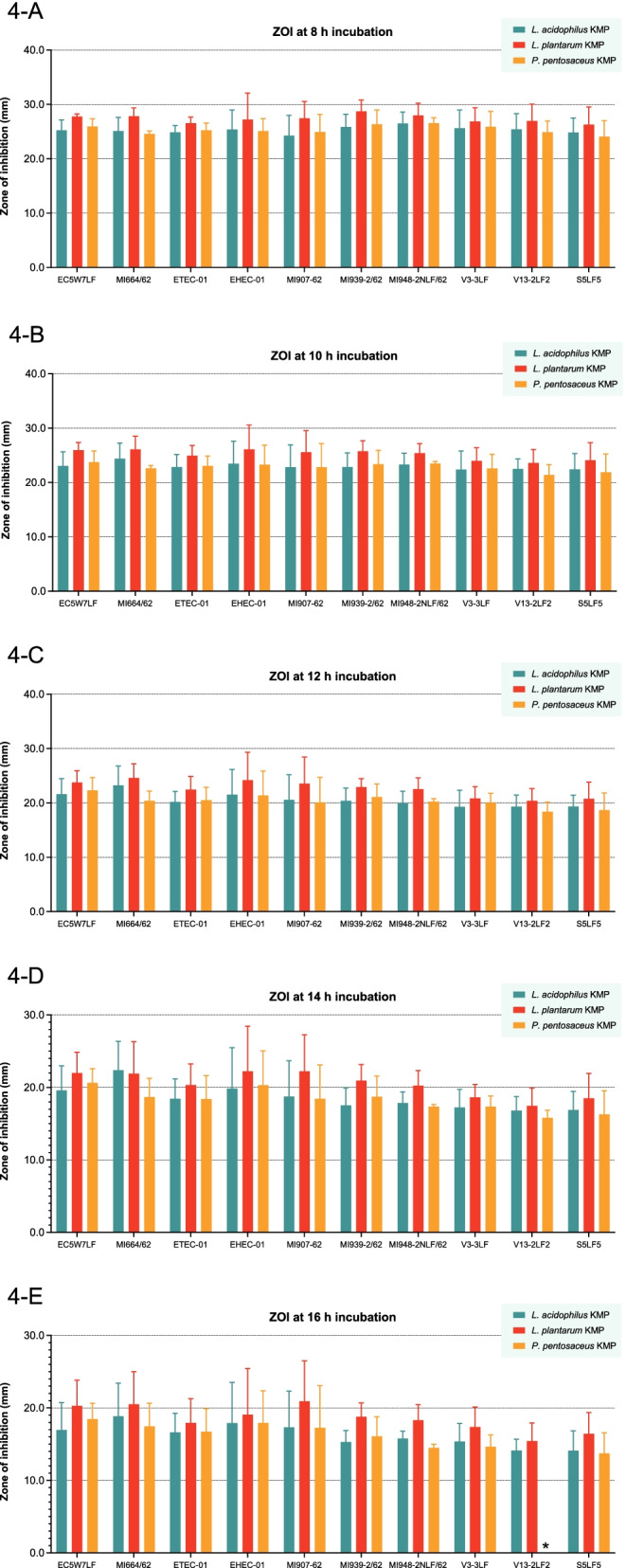
The Comparison of ZOI between *L. acidophilus* KMP, *L. plantarum* KMP, and *P. pentosaceus* KMP. **A** The presence of ZOI at 8 h incubation. **B** The presence of ZOI at 10 h incubation. **C** The presence of ZOI at 12 h incubation. **D** The presence of ZOI at 14 h incubation. **E** The presence of ZOI at 16 h incubation

The comparison ZOI between *L. acidophilus* KMP, *L. plantarum* KMP, and *P. pentosaceus* KMP at the same point of incubation time with all strains of pathogenic *E. coli* showed in Table 1 and Fig. 4. There were highly significant differences (*P*-value < 0.01) by Duncan’s test at 8 h incubation (Fig. 4A), at 10 h incubation (Fig. 4B), and at 12 h incubation (Fig. 4C). Furthermore, there was statistically significant differences (*P*-value < 0.05) by Duncan’s test at 14 h incubation (Fig. 4D). In details, the ZOI of CFCS from *L. plantarum* KMP showed the highest value at 8, 10, 12, and 14 h incubation. Whereas the ZOI of CFCS from *L. acidophilus* KMP showed the least value at 8, 12, 14 h incubation. Moreover, the ZOI of CFCS from *P. pentosaceus* KMP was lowest at 10 h incubation, significantly (*P*-value < 0.05) by Duncan’s test. These results clearly indicated that the CFCS from *L. plantarum* KMP was more efficient in inhibiting the growth of pathogenic *E. coli* compared with CFCS from *L. acidophilus* KMP and *P. pentosaceus* KMP.

**Table 1:** The comparison of the presence of ZOI (mm) between *L. acidophilus* KMP, *L. plantarum* KMP, and *P. pentosaceus* KMP in various hours of incubation performed using one-way analysis of variance (ANOVA) and compared means by using Duncan’s test

LAB-CFCS	The presence of ZOI (mm) which expressed as mean (*n* = 4)				
	8 h incubation	10 h incubation	12 h incubation	14 h incubation	16 h incubation
*L. acidophilus* KMP	25.31 ± 0.61^b^	23.00 ± 0.61^b^	20.56 ± 1.27^b^	18.56 ± 1.72^b^	16.24 ± 1.59
*L. plantarum* KMP	27.38 ± 0.72^a^	25.16 ± 0.95^a^	22.60 ± 1.50^a^	20.45 ± 1.73^a^	18.50 ± 1.80
*P. pentosaceus* KMP	25.36 ± 0.78^b^	22.82 ± 0.73^b^	20.33 ± 1.16^b^	18.22 ± 1.56^b^	14.69 ± 5.40
***P*-value**	**0.000**	**0.000**	**0.001**	**0.013**	**0.059**

Note: ^(a,b)^ The mean values with different superscript letters were statistically significant (*P*-value < 0.05, Duncan’s test)

### The acid and bile tolerance test

**Fig. 5:**
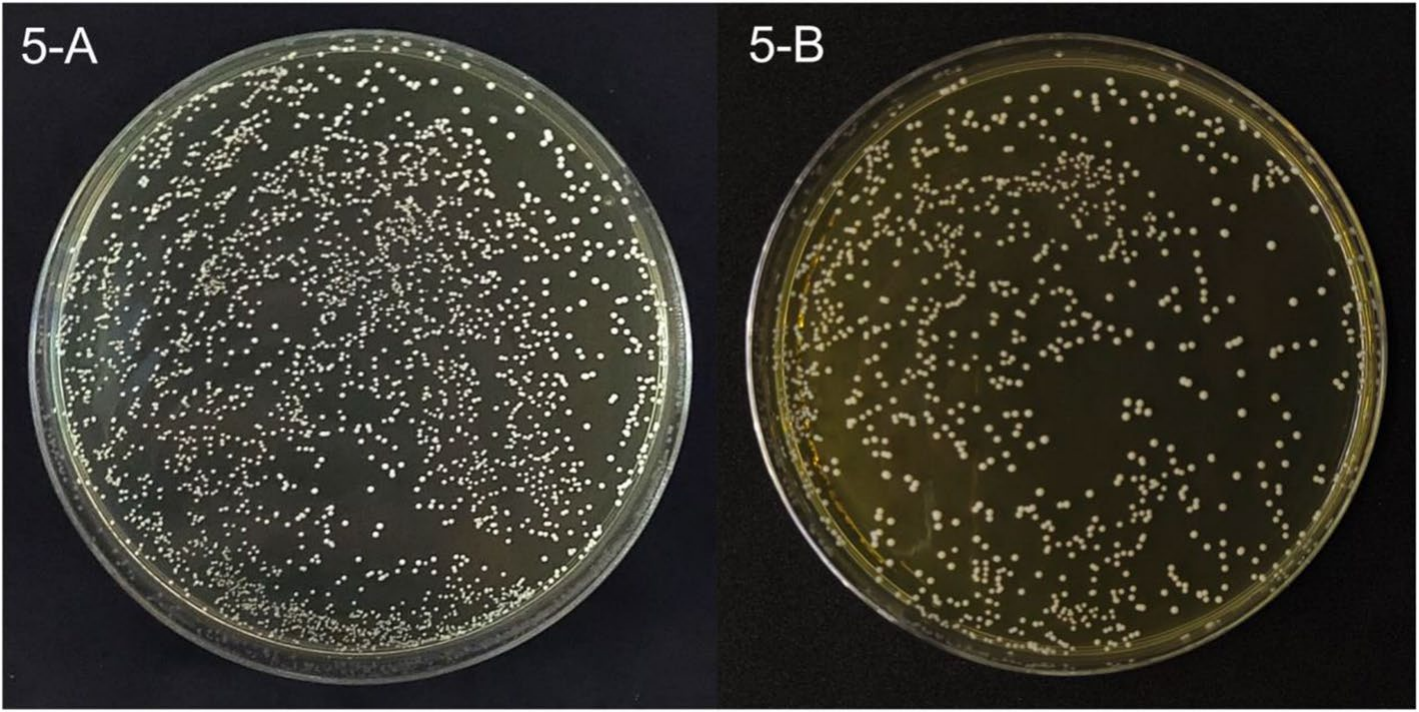
The bile tolerance test showed the viable colonies. **A** The viable colonies of *L. plantarum* KMP in estimated dilution 10^2^ CFU/mL with the condition of 0.3% (w/v) bile salt, observed at 24 h incubation. **B** The viable colonies of *P. pentosaceus* CU115 in estimated dilution 10^2^ CFU/mL with the condition of 0.3% (w/v) bile salt, observed at 24 h incubation

To evaluate the viability of LAB under various environmental condition, the acid and bile tolerance test was performed in this study. The bacteria survival rate was calculated at 3 h incubation. The results of acid and bile salt tolerant abilities of LAB were presented in Fig. 5 and Tables 2, 3, and 4.

**Table 2:** The bacteria survival rate of *L. plantarum* KMP in MRSC broth at 37 ^o^C for 0 and 3 h incubation under the acid condition (pH 2.0 and pH 3.0) and bile salt condition 0.3 and 0.5% (w/v)

***L. plantarum*** KMP	0 h incubation			3 h incubation		
	Viable bacterial count (CFU/mL)	Viable bacterial count (log CFU/mL)	Bacteria survival rate (%)	Viable bacterial count (CFU/mL)	Viable bacterial count (log CFU/mL)	Bacteria survival rate (%)
**Acid tolerance**						
pH 2.0	3.30 × 10^5^	5.52	100	0	–	0
pH 3.0	3.50 × 10^5^	5.54	100	3.10 × 10^5^	5.49	89
Control	2.70 × 10^5^	5.43	100	1.10 × 10^6^	6.04	407
**Bile tolerance**						
0.3%	3.70 × 10^5^	5.57	100	1.49 × 10^6^	6.17	403
0.5%	3.00 × 10^5^	5.48	100	1.48 × 10^6^	6.17	493
Control	4.70 × 10^5^	5.67	100	1.35 × 10^6^	6.13	287

**Table 3:** The bacteria survival rate of *L. acidophilus* KMP in MRSC broth at 37 ^o^C for 0 and 3 h incubation under the acid condition (pH 2.0 and pH 3.0) and bile salt condition 0.3 and 0.5% (w/v)

***L. acidophilus*** KMP	0 h incubation			3 h incubation		
	Viable bacterial count (CFU/mL)	Viable bacterial count (log CFU/mL)	Bacteria survival rate (%)	Viable bacterial count (CFU/mL)	Viable bacterial count (log CFU/mL)	Bacteria survival rate (%)
**Acid tolerance**						
pH 2.0	4.10 × 10^5^	5.61	100	0	–	0
pH 3.0	6.40 × 10^5^	5.81	100	1.59 × 10^6^	6.20	248
Control	5.90 × 10^5^	5.77	100	1.45 × 10^6^	6.16	246
**Bile tolerance**						
0.3%	5.00 × 10^5^	5.70	100	1.43 × 10^6^	6.16	286
0.5%	4.60 × 10^5^	5.66	100	6.70 × 10^5^	5.83	146
Control	3.00 × 10^5^	5.48	100	1.58 × 10^6^	6.20	527

**Table 4:** The bacteria survival rate of *P. pentosaceus* KMP in MRSC broth at 37 ^o^C for 0 and 3 h incubation under the acid condition (pH 2.0 and pH 3.0) and bile salt condition 0.3 and 0.5% (w/v)

***P. pentosaceus*** KMP	0 h incubation			3 h incubation		
	Viable bacterial count (CFU/mL)	Viable bacterial count (log CFU/mL)	Bacteria survival rate (%)	Viable bacterial count (CFU/mL)	Viable bacterial count (log CFU/mL)	Bacteria survival rate (%)
**Acid tolerance**						
pH 2.0	3.00 × 10^4^	4.48	100	0	–	0
pH 3.0	6.30 × 10^4^	4.80	100	3.00 × 10^4^	4.48	48
Control	1.80 × 10^5^	5.26	100	1.68 × 10^6^	6.23	933
**Bile tolerance**						
0.3%	1.70 × 10^5^	5.23	100	4.60 × 10^5^	5.66	271
0.5%	2.00 × 10^5^	5.30	100	6.10 × 10^5^	5.79	305
Control	5.00 × 10^5^	5.70	100	3.36 × 10^6^	6.53	672

*L. acidophilus* KMP, *L. plantarum* KMP, and *P. pentosaceus* KMP showed no viability in acidic condition of pH 2.0. On the contrary, they exhibited viability in pH 3.0 with bacteria survival rate 248, 89, and 48% respectively. In bile tolerance test, *L. acidophilus* KMP, *L. plantarum* KMP, and *P. pentosaceus* KMP survived well in both of 0.3 and 0.5% bile salt concentration. *L. plantarum* KMP presented greatest value of bacteria survival rate at 403% in 0.3% bile salt concentration (Table 3), while *L. acidophilus* and *P. pentosaceus* KMP exhibited in 286 and 271% respectively (Tables 2 and 4). There was the same tendency in 0.5% bile salt concentration. *L. plantarum* KMP presented greatest value of bacteria survival rate at 493% as well (Table 2).

Together, the results were possibly implied that *L. acidophilus* KMP, *L. plantarum* KMP, and *P. pentosaceus* KMP could tolerate in pH 3.0, 0.3 and 0.5% bile salt concentration. *L. acidophilus* KMP showed the most tolerant property in acidic environment when compared with *L. plantarum* KMP and *P. pentosaceus* KMP in the same acidic condition and time. On the contrary, *L. plantarum* KMP exhibited the greatest value of bacteria survival rate in bile salt conditions when compared with *L. acidophilus* KMP and *P. pentosaceus* KMP in the same bile salt concentrations and time.

### Cross-streaking assay

**Fig. 6:**
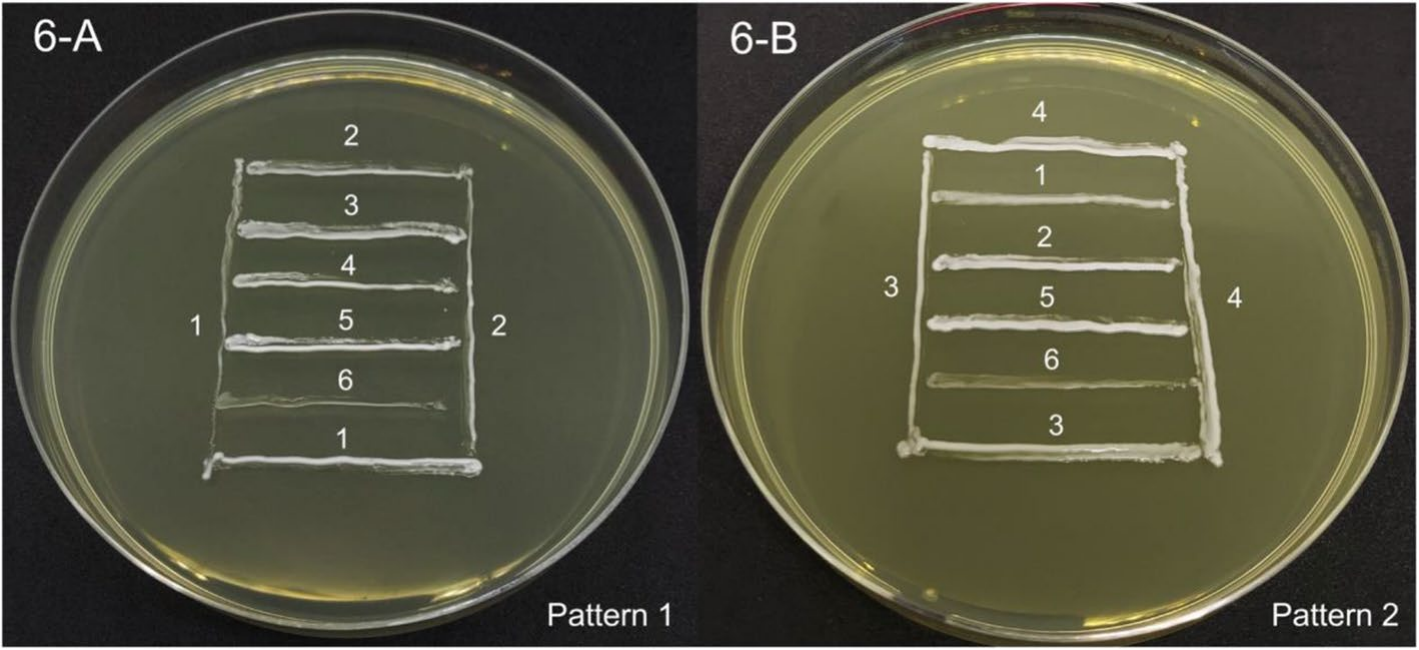
The cross-streaking assay showed the absence of ZOI among 6 strains of LAB-streaked lines on MRSC solid agar. **A** Pattern 1 of LAB-streaked lines. **B** Pattern 2 of LAB-streaked lines. (1) = *L. acidophilus* KMP, (2) = *L. plantarum* KMP, (3) = *P. pentosaceus* KMP, (4) = *L. plantarum* CU31-5B, (5) = *P. pentosaceus* CU115, and (6) = *E. faecium* CU28-1 M

To study the antagonist activities of LAB, 6 strains of LAB were streaked out in the same media plate. The remarkable criteria to evaluate were the presence or absence inhibitory zone between close contact points of LAB-streaked lines. The results were presented in Fig. 6 and Table 5.

**Table 5:** The study of antagonist activities among 6 strains of LAB by cross-streaking assay

LAB in study	*L. acidophilus* KMP	*L. plantarum* KMP	*P. pentosaceus* KMP	*L. plantarum* CU31-5B	*P. pentosaceus* CU115	*E. faecium* CU28-1 M
*L. acidophilus* KMP		–	–	–	–	–
*L. plantarum* KMP	–		–	–	–	–
*P. pentosaceus* KMP	–	–		–	–	–
*L. plantarum* CU31-5B	–	–	–		–	–
*P. pentosaceus* CU115	–	–	–	–		–
*E. faecium* CU28-1 M	–	–	–	–	–	

Note: The criteria of interpretation are marked by the following: (−) = absence of ZOI, (+) = presence of ZOI diameter 1–3 mm, (++) = presence of ZOI diameter 4–6 mm, (+++) = presence of ZOI diameter 7–9 mm, (++++) = presence of ZOI diameter > 9 mm

*L. acidophilus* KMP, *L. plantarum* KMP, *P. pentosaceus* KMP, *L. plantarum* CU31-5B, *P. pentosaceus* CU115, and *E. faecium* CU28-1 M exhibited no inhibitory zone within close contact point of the streaked lines. Thus, this result indicated no antagonist activities among all strains of LAB.

## Discussion

The present results on agar well diffusion assay clearly showed that the CFCS from *L. acidophilus* KMP, *L. plantarum* KMP, and *P. pentosaceus* KMP could inhibit the growth of 10 strains of pathogenic *E. coli* which were isolated from pigs in Thailand. However, the ZOI could be reversible depending on time of incubation and had a wide range of diameter compared to the previous studies [34–36]. According to Lin et al. [34], the ZOI inhibitory activity of the CFCS from *L. acidophilus* RY2 against enteroaggregative *E. coli* strains at 14 h incubation could be divided into 3 size-groups: 11–16 mm (small), 17–22 mm (medium), more than 23 mm (large). Comparing with this work results of *L. acidophilus* at the same incubation time, the ZOI of CFCS from *L. acidophilus* KMP against pathogenic *E. coli* was varied from 17.5 ± 2.5 to 22.2 ± 6.2 mm diameter which showed the inhibitory effect between medium and large size. In contrast to our results of *L. plantarum*, Mao et al. [36] reported that the ZOI of CFCS from LAB such as *L. plantarum* DY1, *L. plantarum* DY6, and *L. plantarum* DY7 had antibacterial activities on *E. coli* ATCC25922, the ZOI from *L. plantarum* DY1, *L. plantarum* DY6, and *L. plantarum* DY7 were 12.89 ± 0.21, 15.32 ± 0.28, and 13.79 ± 0.33 mm diameter respectively, comparing to our study, the ZOI of CFCS from *L. plantarum* KMP against pathogenic *E. coli* at 16 h incubation was varied from 15.4 ± 2.5 to 20.9 ± 5.6 mm diameter.

Similar to our results on the ZOI of CFCS from *P. pentosaceus* KMP against pathogenic *E. coli*, Bajpai et al. [35] reported the ZOI of CFCS from *P. pentosaceus* 4I1 against both Gram-positive and Gram-negative bacteria including *E. coli* O157:H7 with a range of 16.5–20.4 mm diameter, compared to the ZOI of CFCS from *P. pentosaceus* KMP against pathogenic *E. coli* of 13.7 ± 2.8 to 18.5 ± 2.2 mm diameter at 16 h incubation in our study. However, the largest ZOI of CFCS from *P. pentosaceus* KMP against pathogenic *E. coli* was varied from 24.1 ± 2.9 to 26.5 ± 1.0 mm diameter at 8 h incubation which was larger than the inhibitory effect reported by Bajpai et al. [35].

In the present study, the maximal diameter ZOI of CFCS was found at 8 h incubation and thereafter gradually decreased from 10 h incubation until no inhibitory zone was observed at 18 h incubation. The reason might be that at 18 h incubation, the concentration of the active component in CFCS that promoted the inhibition was diminished [37]. Our results might also be explained by previous study from Garg et al. [29] in that the decreased activity of CFCS while the increased CFU at each point of incubation time showed the bacteriostatic effect of CFCS, in which, could be hypothesized that the action of CFCS might be neutralized by the metabolic end products produced by pathogenic bacteria strain. To the best of our knowledge, there are no experiment of the re-added the CFCS into the agar well when the ZOI was absence to evaluate the concentration-depended manner of the CFCS against pathogenic *E. coli*. Therefore, further study is needed to prove this hypothesis.

The CFCS from the LAB such as *Lactobacillus* spp., *Pediococcus* spp., or *Enterococcus* spp. were reported to produce many active components including organic acids, hydrogen peroxide (H_2_O_2_), protein and diacetyl [31, 37]. Hartmann et al. [27] also stated that the different active substances in CFCS work synergistically with each other, indicating that the CFCS has an over advantage than purified antimicrobial components. To support the synergistic effect of CFCS active components, Zheng et al. [38] carried out the comparative experiment between buffer with the similar pH to CFCS and CFCS from *E. faecium*. The study showed that the inhibitory zone of CFCS was significantly larger than the inhibitory zone from buffer with the similar pH to CFCS. In addition, Tenea and Barrigas [24] reported that the CFCS from *L. plantarum* Cys5–4 contained the bacteriocins, but the inhibitory effects differed between CFCS and precipitated protein for Gram-negative bacteria including *E. coli* significantly, suggesting that the organic acid in CFCS may exerted the action than only precipitated protein. In different points of view, Koohestani et al. [31] demonstrated that the antibacterial activity of *L. acidophilus* LA-5 was not related to bacteriocin, but mainly related to lactic acid production. This could be speculated that not all of LAB can produce AMPs. To support antimicrobial role of acidic compotents, Qian et al. [26] demonstrated that the acidity of CFCS was of significant for AMPs activity of CFCS in that the neutralized CFCS had a lower reduction in microbial growth than CFCS with acidic pH. By the other sides, Hassen et al. [39] has been demonstrated that LAB can produce a group of AMPs, including bacteriocins, which have potential to kill or inhibit the growth of pathogens. To support this hypothesis, it has been reported that *L. paracasei* subsp. *toleran*s FX-6 from Tibetan kefir can produce a novel AMP, namely bacteriocin F1 with a molecular weight of 2–5 kDa [40]. As a result of protein or peptide constituents in CFCS, the results from this study confirmed that the CFCS from *L. acidophilus* KMP, *L. plantarum* KMP and *P. pentosaceus* KMP had an efficacy to inhibit growth of 10 strains of pathogenic *E. coli*. However, the CFCS produced by LAB in this study will be further analysis to prove an existence of AMPs. Together, these literatures indicated that there are many active components work together in CFCS and their play a major role in antimicrobial activities. To focus on AMPs efficacy, the non-protein components should be ruled out from the CFCS. To exemplify, Gaspar et al. [41] stated the ways to eliminate antimicrobial effects from organic acid by adjusting the pH to same MRSC broth (pH 6.5) and from the hydrogen peroxide by adding the catalase. However, the CFCS from our study were not tested and proved by using mentioned conditions. Thus, the further study is recommended eliminating or neutralizing other active compounds to validate the inhibitory activities.

In the digestive system of healthy pigs, there were protective mechanisms for pathogens such as low pH gastric juice and proteolytic enzymes in the stomach or bile salts in the intestine. Thus, one of the criteria for probiotics selection was acid and bile tolerance. This was the major factor for probability of probiotics and its active components of CFCS survival in pig’s gastrointestinal tract. Dowarah et al. [42] reported that the resistance to pH of the expected component must be lower than pH 3.0 and remain viable in the gastric region for 4 h or more due to the acidity of the stomach. However, Wang et al. [43] reported that the ability to tolerate the environment of the gastrointestinal tract with the pH of pig gastric juices being as low as 2.0 and bile with pH of about 8.0. In addition, Hatton et al. [44] investigated the post-mortem pigs and found that the mean gastric pH was 4.4 when fed ad libitum and pH between 6.1–6.7 in small intestines. It was well documented that *Lactobacilli* were bile and acid tolerance and can survive more than the other LAB as well as they were not pathogenic [45]. It was worth noting that all LAB in the present study show ability of acid and bile tolerance. Thus, feeding the pigs with probiotics and its CFCS that showed tolerance to acid and bile might be the alternative way to feed the pigs to improve their gastrointestinal function.

Together, the result from agar well diffusion assay found that the ZOI was decreased during 14 to 16 h incubation of time. Thus, the application of usage of CFCS or AMPs for the pigs should be administrated at least every 12 h interval to maintain the antimicrobial efficacy. Otherwise, the future experiment is needed to determine the accuracy time to feed the pigs with CFCS. Furthermore, the tolerance test and the cross-streaking assay figured out the viability of LAB in acidic and bile salt conditions without antagonist properties. Thus, *L. acidophilus* KMP, *L. plantarum* KMP, and *P. pentosaceus* KMP can be utilized as a mixed-probiotics feed additives by oral administration.

## Conclusions

In conclusion, the CFCS from 3 strains of LAB consisting of *L. acidophilus* KMP, *L. plantarum* KMP and *P. pentosaceus* KMP inhibit growth of pathogenic *E. coli* isolated from pigs in Thailand. The antimicrobial activity observed was incubation time dependent. Nevertheless, an existence of AMPs and re-adjusted CFCS conditions need to be investigated in further study. Additionally, *L. acidophilus* KMP, *L. plantarum* KMP and *P. pentosaceus* KMP can tolerate to acid and bile salt and show no antagonistic effect among each other. Ultimately, these can be practically applied as the alternative way instead of antibiotics usage to promote gut health and inhibit pathogenic *E. coli* in pig industry worldwide.

## Methods

This research project was approved by the Faculty of Veterinary Science-Animal Care and Use Committee (FVS-ACUC-Protocol No. MUVS-2019-06-31).

### Culture media and reagents preparation

De Man, Rogosa, and Sharpe (MRS) medium (BD, Franklin Lakes, NJ, USA) with 0.2% L-Cysteine Hydrochloride Monohydrate (TCI, Tokyo, Japan) was combined as De Man, Rogosa, and Sharpe with L-Cysteine (MRSC) and used for lactic acid producing bacteria (LAB) culture [46]. Additionally, MRSC broth was added to purified 1 N hydrochloric acid (HCl) and bile salt to adjust acidity and set up the concentration of bile experimental conditions in tolerance tests [47]. Brian Heart Infusion (BHI) broth (Oxoid, Hampshire, UK) was used to cultivate fastidious microorganisms, *E. coli*. Nutrient agar (NA) (Oxoid, Hampshire, UK) was used in agar well diffusion assay [30].

### Preparation of LAB strains

Culture stocks of the 3 LAB strains including *L. plantarum* KMP, *L. acidophilus* KMP*,* and *P. pentosaceus* KMP were obtained from a private company (K.M.P. Biotech Co., Ltd., Chonburi, Thailand). The other LAB strains, including *Pediococcus*, and *Enterococcus*, were obtained from the culture collection of Bacterial Laboratory, Veterinary Diagnostic Center, Faculty of Veterinary Science, Mahidol University. Briefly, all LAB (Table 6) were preserved and stored as a in glycerol at -80 °C. The *Lactobacillus* spp., *Pediococcus*, and *Enterococcus* were streaked onto MRSC agar, incubated at 37 °C under aerobic condition overnight. Then, the colonies of *Lactobacillus* spp., and *Pediococcus* were inoculated into MRSC broth and grown at 37 °C with shaking overnight and stored at 4 °C until used [48, 49].

**Table 6:** LAB strain used in this study as stock culture from K.M.P. Biotech Co., Ltd., and the Faculty of Veterinary Science, Mahidol University (MUVS), Thailand

Bacteria used	Strain code	Abbreviation	Origin
*Lactobacillus acidophilus*	KMP	LA-KMP	K.M.P. Co., Ltd.
*Lactobacillus plantarum*	KMP	LP-KMP	K.M.P. Co., Ltd.
*Pediococcus pentosaceus*	KMP	PP-KMP	K.M.P. Co., Ltd.
*Lactobacillus plantarum*	CU31-5B	LP-CU	MUVS stock culture
*Pediococcus pentosaceus*	CU115	PP-CU	MUVS stock culture
*Enterococcus faecium*	CU28-1 M	EF-CU	MUVS stock culture

### Preparation of pathogenic *E. coli* strains

Clinical isolates of 10 different strains of pathogenic *E. coli* were collected from clinical signs presenting pigs in Thailand (Table 7). Virulence factors from each strain of pathogenic *E. coli* are identified of genes encoding for fimbriae and toxins by polymerase chain reaction (PCR). Then, the pathogenic *E. coli* were preserved and stored with glycerol in -80 °C by following the ATCC guideline [50] as stock culture collection of Bacterial Laboratory, Veterinary Diagnostic Center, Faculty of Veterinary Science, Mahidol University. Briefly, the pathogenic *E. coli* were streaked onto MacConkey agar (Clinag Co., Ltd., Bangkok, Thailand) and incubated at 37 °C under aerobic condition overnight. Then, the colonies of pathogenic *E. coli* were inoculated into BHI broth and grown at 37 °C with shaking overnight and stored at 4 °C until used [25].

**Table 7:** The 10 strains of pathogenic *E. coli* as clinical isolation from pigs in Thailand

Bacterial strains	Description / Virulence factor	Reference
***Escherichia coli***		
EC5W7LF	Clinical isolation from pig feces / F4 fimbriae	MUVS stock culture
MI664/62	Clinical isolation from pig feces / F18 fimbriae	MUVS stock culture
ETEC-01	Clinical isolation from pig feces	MUVS stock culture
EHEC-01	Clinical isolation from pig feces	MUVS stock culture
MI907–62	Clinical isolation from pig feces / F4 fimbriae	MUVS stock culture
MI939–2/62	Clinical isolation from pig feces / Shiga toxin type 1 (*stx-1*) gene	MUVS stock culture
MI948-2NLF/62	Clinical isolation from pig feces / F18 fimbriae	MUVS stock culture
V3-3LF	Clinical isolation from pig vaginal swab / Shiga toxin type 1 (*stx-1*) gene, *escV* gene	MUVS stock culture
V13-2LF2	Clinical isolation from pig vaginal swab	MUVS stock culture
S5LF5	Clinical isolation from pig semen / *astA* gene, *int1* gene	MUVS stock culture

### Cell-free culture supernatant (CFCS) preparation

The following protocol was modified from previous studies [28, 48, 49]. *Lactobacillus* spp., and *Pediococcus* were inoculated in MRSC broth and grown at 37 °C with shaking overnight. Then, the inoculated MRSC broth was transferred to 1.5 mL microcentrifuge tube and performed centrifugation for 2 min at 3578×*g* (Denville Micro 260D Microcentrifuge, Denville Scientific, Inc., Metuchen, NJ, USA). Supernatant was collected by sterile syringe with needle and then filtered with a sterile polyethersulfone (PES) membrane filter with pore size 0.22 μm (Guangzhou Jet Bio-Filtration Co., Ltd., Guangzhou, China). Thereafter, the CFCS was used freshly in agar well diffusion assay and stored in -20 °C for further study, more details were provided (see Additional file 2).

### Agar well diffusion assay

The agar well diffusion assay aimed to evaluate the inhibitory activities of LAB-CFCS against pathogenic *E. coli*. This method was adapted from Bajpai et al. [35]. Initially, nutrient agar plates were initially inoculated with 0.5 McFarland standard (10^8^ cells/mL) of pathogenic *E. coli*. A sterile 8 mm diameter cork borer was used to pierce wells into nutrient agar. 80 μL of LAB-CFCS were loaded in each well, and the plates were incubated at 37 °C under aerobic condition. Furthermore, sterile MRSC broth was loaded as a negative control to validate the experiment. To evaluate the efficiency of CFCS, the measurement of ZOI diameter in millimeters (mm) was performed repeatedly at 8, 10, 12, 14, and 16 h of incubation. Further details were provided (see Additional file 2).

### The acid and bile tolerance test

This experiment was performed to evaluate the viability of LAB under the environmental condition present in the swine gastrointestinal tract. The assay was adapted from Hassanzadazar et al. [20]. MRSC medium was modified according to experimental conditions. There were 3 conditions for each tolerance test. The acid tolerance was determined at pH 2.0 and pH 3.0 by adjusting pH of MRSC broth with 1 N HCl. The bile tolerance was determined by adding bile salt 0.3% (w/v) and 0.5% (w/v) to MRSC broth. *Lactobacillus* spp. and *Pediococcus* spp. overnight cultures were adjusted to 0.5 McFarland standard (10^8^ cells/mL) in 0.9% sterile normal saline solution (NSS). Then, 10-fold serial dilution was performed for the final concentration at 10^2^ cells/mL. One hundred microliters of each dilution were inoculated in MRSC broth at different pH and bile salt concentration, at 37 °C, with shaking at 200 RPM. Then, the inoculated MRSC broth in each condition was collected at 0 and 3 h and performed spread plate method onto MRSC plate. The plates were incubated at 37 °C and colonies were counted at 24 h incubation. MRSC broth with the LAB inoculation was used as positive control, and sterile MRSC broth as the negative control. The percentage of bacteria survival rate was calculated using the following equation adapted from Guan et al. [47]:

$$Bacteria\ survival\ rate\ \left(\%\right)=\frac{CFU\ assay\ast }{CFU\ control}\times 100$$

Note: *CFU assay is the cell count after incubation in each time and CFU control is the cell count after 0 h incubation in positive control.

### Cross-streaking assay

This experiment aimed to evaluate antagonistic activities among 6 strains of LAB. There were modified the principle and criteria of result interpretation from previous studies [51, 52]. The antagonistic activities were presented by an inhibitory zone among LAB-streaked lines. Although there were many effective methods to evaluate antagonistic activities, the cross-streaking assay was selected because of its simplicity, and rapid method in screening culture [53]. Nevertheless, the indistinct and unclear inhibitory zone were mentioned as major limitation [53, 54]. According to results reliability, the experiment was performed in triplicate. Firstly, single colony from 3 strains of *Lactobacillus* spp. and other 3 LAB (*P. pentosaceus* and *E. faecium*) was picked up and streaked onto MRSC plate. The streaked lines were designed for 3 different patterns to completely test all strains used LAB, more details were provided (see Additional file 2). Then, the plates were incubated at 37 °C under anaerobic condition for 24 h.

### Statistical analysis

Descriptive statistics were used in this study. All the data were expressed as mean ± standard deviation (SD) performed using the Microsoft Excel 365 on Mac (Microsoft Corporation, Redmond, WA, USA). The figures were illustrated by the Prism version 9.3.0 on Mac (GraphPad Software, San Diego, CA, USA). The data analysis was performed by using one-way analysis of variance (ANOVA) and compared means by using Duncan’s test by SPSS version 25.0 on Mac (SPSS Inc., Chicago, IL, USA). A statistical significance is determined as *P*-value < 0.05.

## Supplementary Information

**Additional file 1: Table S1.** The study of inhibitory activities of CFCS produced from *L. acidophilus* KMP against 10 strains of pathogenic *E. coli* isolated from swine, study via agar well diffusion assay in NA solid media with various time of incubation. Data are express as mean ± S.D. (*n* = 4). **Table S2.** The study of inhibitory activities of CFCS produced from *L. plantarum* KMP against 10 strains of pathogenic *E. coli* isolated from swine, study via agar well diffusion assay in NA solid media with various time of incubation. Data are express as mean ± S.D. (*n* = 4). **Table S3.** The study of inhibitory activities of CFCS produced from *P. pentosaceus* KMP against 10 strains of pathogenic *E. coli* isolated from swine, study via agar well diffusion assay in NA solid media with various time of incubation. Data are express as mean ± S.D. (*n* = 4).

**Additional file 2: Figure S1.** The Cell-free culture supernatant preparation procedures, the cultivation of LAB in MRSC broth were inoculated overnight (1-A). Centrifugation process of LAB broth (1-B), Contents in sample tube separated into two parts supernatant (liquid part) and precipitation after centrifugation (1-C). Supernatant was drained out into sterile syringe (1-D). The filtration process was performed with sterile PES membrane (1-E). The CFCS was completely prepared and ready to use (1-F). **Figure S2.** The demonstration of agar well diffusion assay. **Figure S3.** Agar well diffusion assay, the presence of ZOI from CFCS against pathogenic *E. coli* V13-2LF2 at 8 h incubation (3-A). Meanwhile, the presence of ZOI from CFCS was significantly decreased at 16 h incubation (3-B). **Figure S4.** The cross-streaking assay, an illustration of 3 different patterns: pattern 1 (4-A), pattern 2 (4-B), and pattern 3 (4-C) in experiment and demonstration of 6 streaked lines of LAB. Close contact points are marked as a black arrow.

## Data Availability

The datasets used and/or analysed during the current study available from the corresponding author on reasonable request.

## Abbreviations

AMPs: Antimicrobial peptides
BHI: Brain heart infusion medium
CFCS: Cell-free culture supernatants
CFU: Colony forming unit
EPEC: Enteropathogenic *Escherichia coli*
ETEC: Enterotoxigenic *Escherichia coli*
LAB: Lactic Acid Bacteria
MRS: De Man, Rogosa, and Sharpe medium
MRSC: De Man, Rogosa, and Sharpe with L-Cysteine medium
NA: Nutrient agar medium
PCR: polymerase chain reaction
PES: Polyethersulfone
PWD: Post-weaning diarrhea
ZOI: Zone of inhibition
