# Supplementary material for: Cell-free culture supernatants of Lactobacillus spp. and Pediococcus spp. inhibit growth of pathogenic Escherichia coli isolated from pigs in Thailand
Source: BMC Vet Res. 2022 Jan 29;18:60. doi: 10.1186/s12917-022-03140-8 (PMC8800250; doi:10.1186/s12917-022-03140-8)
Supplement: Supplementary file 1 — Additional file 1: Table S1. The study of inhibitory activities of CFCS produced from L. acidophilus KMP against 10 strains of pathogenic E. coli isolated from swine, study via agar well diffusion assay in NA solid media with various time of incubation. Data are express as mean ± S.D. (n = 4). Table S2. The study of inhibitory activities of CFCS produced from L. plantarum KMP against 10 strains of pathogenic E. coli isolated from swine, study via agar well diffusion assay in NA solid media with various time of incubation. Data are express as mean ± S.D. (n = 4). Table S3. The study of inhibitory activities of CFCS produced from P. pentosaceus KMP against 10 strains of pathogenic E. coli isolated from swine, study via agar well diffusion assay in NA solid media with various time of incubation. Data are express as mean ± S.D. (n = 4). [file 12917_2022_3140_MOESM1_ESM.docx]

**Supplementary Table 1:** The study of inhibitory activities of CFCS produced from *L. acidophilus* KMP against 10 strains of pathogenic *E. coli* isolated from swine, study via agar well diffusion assay in NA solid media with various time of incubation. Data are express as mean ± S.D. (n = 4).

| Experiment group | The presence of ZOI (mm) of CFCS produced from *L. acidophilus* KMP in various time of incubation | | | | | |
| --- | --- | --- | --- | --- | --- | --- |
|  | 8 h incubation | 10 h incubation | | 12 h incubation | 14 h incubation | 16 h incubation |
| **Control group** |  |  |  |  |  |  |
| MRSC broth | 0.0 ± 0.0 | 0.0 ± 0.0 | | 0.0 ± 0.0 | 0.0 ± 0.0 | 0.0 ± 0.0 |
| ***Escherichia coli*** |  |  | |  |  |  |
| EC5W7LF | 25.2 ± 1.9 | 23.0 ± 2.6 | | 21.6 ± 2.9 | 19.6 ± 3.4 | 17.0 ± 3.8 |
| MI664/62 | 25.1 ± 2.5 | 24.4 ± 2.9 | | 23.3 ± 3.6 | 22.4 ± 4.0 | 18.9 ± 4.5 |
| ETEC-01 | 24.9 ± 1.3 | 22.8 ± 2.3 | | 20.2 ± 1.9 | 18.5 ± 2.7 | 16.6 ± 2.6 |
| EHEC-01 | 25.4 ± 3.6 | 23.5 ± 4.2 | | 21.5 ± 4.6 | 19.9 ± 5.6 | 17.9 ± 5.6 |
| MI907-62 | 24.3 ± 3.7 | 22.8 ± 4.1 | | 20.6 ± 4.6 | 18.8 ± 4.9 | 17.3 ± 5.0 |
| MI 939-2/62 | 25.9 ± 2.3 | 22.9 ± 2.6 | | 20.4 ± 2.4 | 17.5 ± 2.4 | 15.3 ± 1.6 |
| MI 948-2NLF/62 | 26.5 ± 2.1 | 23.3 ± 2.1 | | 20.0 ± 2.2 | 17.9 ± 1.5 | 15.8 ± 1.0 |
| V3-3LF | 25.6 ± 3.4 | 22.4 ± 3.4 | | 19.3 ± 3.0 | 17.3 ± 2.5 | 15.4 ± 2.5 |
| V13-2LF2 | 25.4 ± 2.9 | 22.5 ± 1.8 | | 19.3 ± 2.1 | 16.8 ± 1.9 | 14.1 ± 1.6 |
| S5LF5 | 24.8 ± 2.7 | 22.4 ± 2.9 | | 19.4 ± 2.1 | 16.9 ± 2.6 | 14.1 ± 2.7 |

**Supplementary Table 2:** The study of inhibitory activities of CFCS produced from *L. plantarum* KMP against 10 strains of pathogenic *E. coli* isolated from swine, study via agar well diffusion assay in NA solid media with various time of incubation. Data are express as mean ± S.D. (n = 4).

| Experiment group | The presence of ZOI (mm) of CFCS produced from *L. plantarum* KMP in various time of incubation | | | | | |
| --- | --- | --- | --- | --- | --- | --- |
|  | 8 h incubation | 10 h incubation | | 12 h incubation | 14 h incubation | 16 h incubation |
| **Control group** |  |  |  |  |  |  |
| MRSC broth | 0.0 ± 0.0 | 0.0 ± 0.0 | | 0.0 ± 0.0 | 0.0 ± 0.0 | 0.0 ± 0.0 |
| ***Escherichia coli*** |  |  | |  |  |  |
| EC5W7LF | 27.8 ± 0.5 | 26.0 ± 1.4 | | 23.7 ± 2.2 | 22.0 ± 2.9 | 20.3 ± 3.6 |
| MI664/62 | 27.8 ± 1.5 | 26.1 ± 2.4 | | 24.6 ± 2.6 | 21.9 ± 4.4 | 20.5 ± 4.5 |
| ETEC-01 | 26.6 ± 1.1 | 24.9 ± 1.9 | | 22.5 ± 2.4 | 20.3 ± 2.9 | 17.9 ± 3.3 |
| EHEC-01 | 27.2 ± 4.9 | 26.1 ± 4.5 | | 24.2 ± 5.1 | 22.2 ± 6.2 | 19.1 ± 6.4 |
| MI907-62 | 27.5 ± 3.1 | 25.6 ± 4.0 | | 23.6 ± 4.9 | 22.2 ± 5.0 | 20.9 ± 5.6 |
| MI 939-2/62 | 28.7 ± 2.1 | 25.8 ± 1.9 | | 22.9 ± 1.5 | 21.0 ± 2.2 | 18.8 ± 1.9 |
| MI 948-2NLF/62 | 28.0 ± 2.2 | 25.4 ± 1.8 | | 22.5 ± 2.1 | 20.3 ± 2.1 | 18.3 ± 2.2 |
| V3-3LF | 26.9 ± 2.5 | 24.0 ± 2.4 | | 20.8 ± 2.2 | 18.6 ± 1.8 | 17.4 ± 2.8 |
| V13-2LF2 | 27.0 ± 3.1 | 23.6 ± 2.4 | | 20.4 ± 2.3 | 17.5 ± 2.5 | 15.4 ± 2.5 |
| S5LF5 | 26.3 ± 3.2 | 24.1 ± 3.2 | | 20.8 ± 3.0 | 18.5 ± 3.4 | 16.4 ± 2.9 |

**Supplementary Table 3:** The study of inhibitory activities of CFCS produced from *P. pentosaceus* KMP against 10 strains of pathogenic *E. coli* isolated from swine, study via agar well diffusion assay in NA solid media with various time of incubation. Data are express as mean ± S.D. (n = 4).

| Experiment group | The presence of ZOI (mm) of CFCS produced from *P. pentosaceus* KMP in various time of incubation | | | | | |
| --- | --- | --- | --- | --- | --- | --- |
|  | 8 h incubation | 10 h incubation | | 12 h incubation | 14 h incubation | 16 h incubation |
| **Control group** |  |  |  |  |  |  |
| MRSC broth | 0.0 ± 0.0 | 0.0 ± 0.0 | | 0.0 ± 0.0 | 0.0 ± 0.0 | 0.0 ± 0.0 |
| ***Escherichia coli*** |  |  | |  |  |  |
| EC5W7LF | 26.0 ± 1.4 | 23.7 ± 2.1 | | 22.3 ± 2.4 | 20.7 ± 1.9 | 18.5 ± 2.2 |
| MI664/62 | 24.6 ± 0.5 | 22.6 ± 0.5 | | 20.4 ± 1.8 | 18.7 ± 2.6 | 17.5 ± 3.2 |
| ETEC-01 | 25.3 ± 1.3 | 23.0 ± 1.8 | | 20.5 ± 2.4 | 18.4 ± 3.2 | 16.7 ± 3.2 |
| EHEC-01 | 25.1 ± 2.3 | 23.3 ± 3.6 | | 21.4 ± 4.5 | 20.3 ± 4.8 | 17.9 ± 4.4 |
| MI907-62 | 24.9 ± 3.2 | 22.8 ± 4.4 | | 20.1 ± 4.6 | 18.5 ± 4.7 | 17.3 ± 5.8 |
| MI 939-2/62 | 26.3 ± 2.6 | 23.4 ± 2.6 | | 21.1 ± 2.4 | 18.7 ± 2.8 | 16.1 ± 2.7 |
| MI 948-2NLF/62 | 26.5 ± 1.0 | 23.5 ± 0.4 | | 20.3 ± 0.5 | 17.4 ± 0.3 | 14.5 ± 0.5 |
| V3-3LF | 25.9 ± 2.8 | 22.6 ± 2.6 | | 20.1 ± 1.7 | 17.4 ± 1.5 | 14.7 ± 1.6 |
| V13-2LF2 | 24.9 ± 2.1 | 21.4 ± 1.9 | | 18.4 ± 1.7 | 15.8 ± 1.1 | 0.0 ± 0.0 |
| S5LF5 | 24.1 ± 2.9 | 21.9 ± 3.3 | | 18.7 ± 3.1 | 16.3 ± 3.3 | 13.7 ± 2.8 |
