# Supplementary material for: Cell-free culture supernatants of Lactobacillus spp. and Pediococcus spp. inhibit growth of pathogenic Escherichia coli isolated from pigs in Thailand
Source: BMC Vet Res. 2022 Jan 29;18:60. doi: 10.1186/s12917-022-03140-8 (PMC8800250; doi:10.1186/s12917-022-03140-8)
Supplement: Supplementary file 2 — Additional file 2: Figure S1. The Cell-free culture supernatant preparation procedures, the cultivation of LAB in MRSC broth were inoculated overnight (1-A). Centrifugation process of LAB broth (1-B), Contents in sample tube separated into two parts supernatant (liquid part) and precipitation after centrifugation (1-C). Supernatant was drained out into sterile syringe (1-D). The filtration process was performed with sterile PES membrane (1-E). The CFCS was completely prepared and ready to use (1-F). Figure S2. The demonstration of agar well diffusion assay. Figure S3. Agar well diffusion assay, the presence of ZOI from CFCS against pathogenic E. coli V13-2LF2 at 8 h incubation (3-A). Meanwhile, the presence of ZOI from CFCS was significantly decreased at 16 h incubation (3-B). Figure S4. The cross-streaking assay, an illustration of 3 different patterns: pattern 1 (4-A), pattern 2 (4-B), and pattern 3 (4-C) in experiment and demonstration of 6 streaked lines of LAB. Close contact points are marked as a black arrow. [file 12917_2022_3140_MOESM2_ESM.docx]

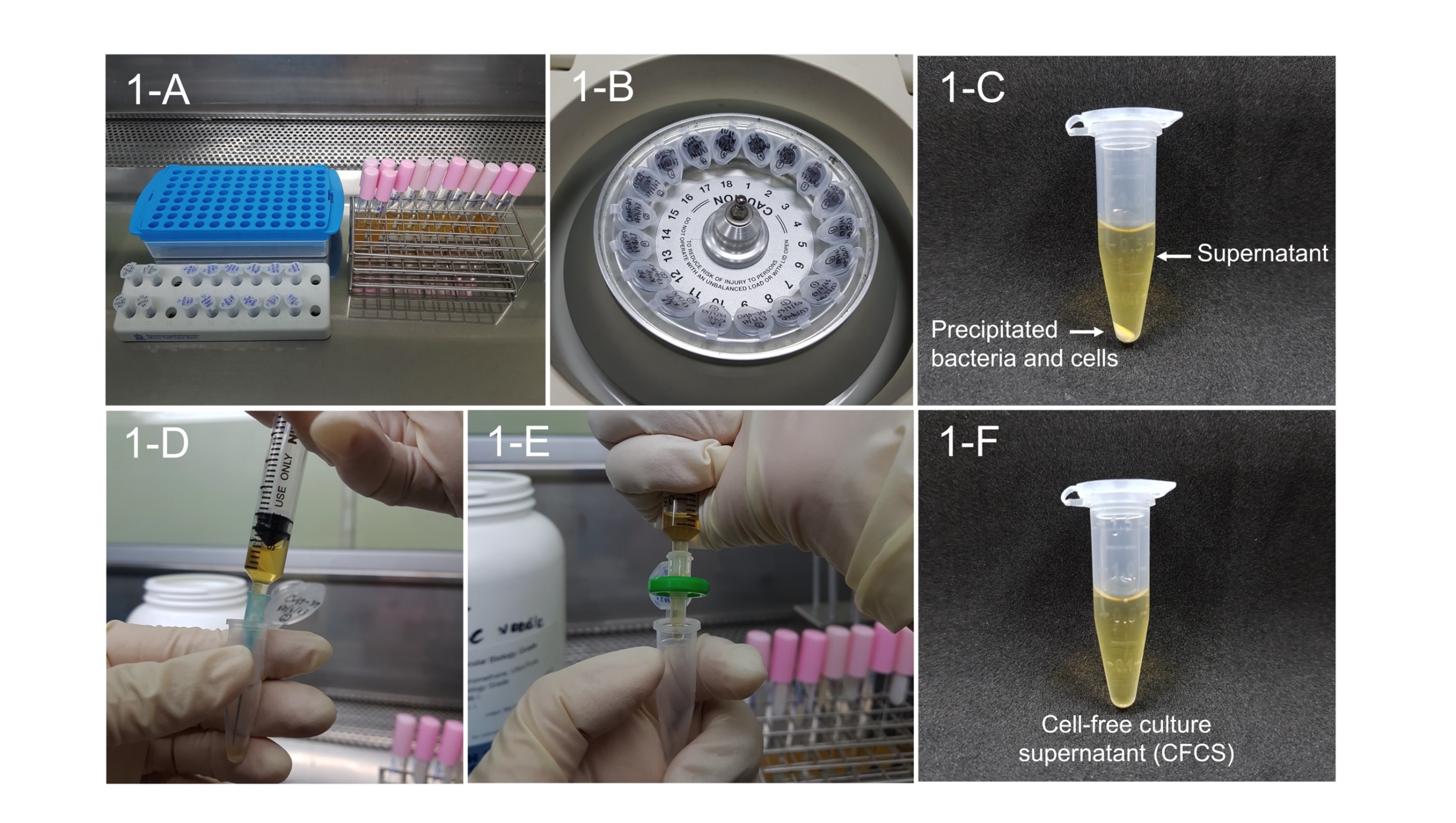


**Supplementary Figure 1:** The Cell-free culture supernatant preparation procedures, the cultivation of LAB in MRSC broth were inoculated overnight (1-A). Centrifugation process of LAB broth (1-B), Contents in sample tube separated into two parts supernatant (liquid part) and precipitation after centrifugation (1-C). Supernatant was drained out into sterile syringe (1-D). The filtration process was performed with sterile PES membrane (1-E). The CFCS was completely prepared and ready to use (1-F).


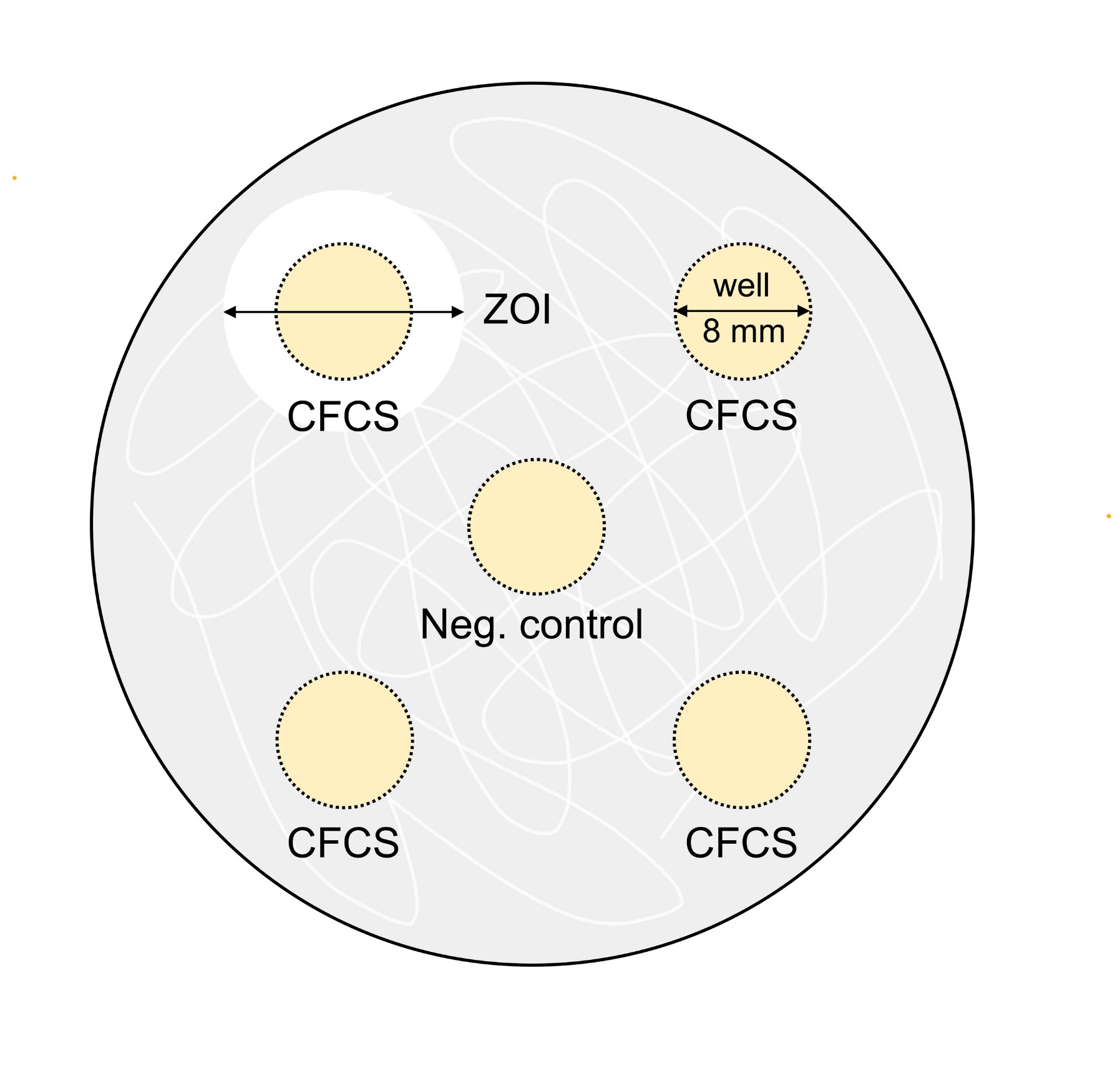


**Supplementary Figure 2:** The demonstration of agar well diffusion assay


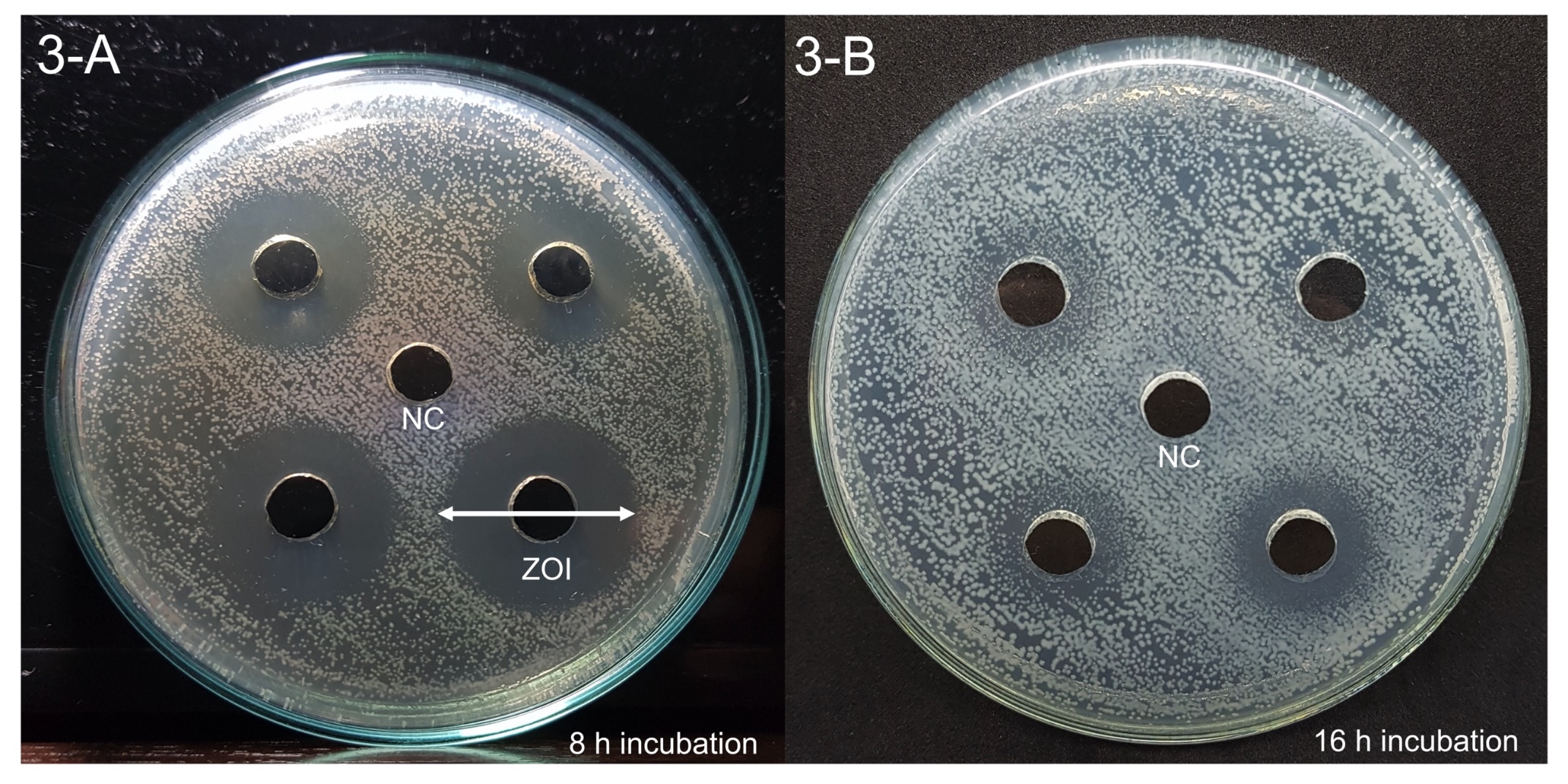


**Supplementary Figure 3:** Agar well diffusion assay, the presence of ZOI from CFCS against pathogenic *E. coli* V13-2LF2 at 8 h incubation (3-A). Meanwhile, the presence of ZOI from CFCS was significantly decreased at 16 h incubation (3-B).

Note: (NC) = Negative control as sterile MRSC broth.


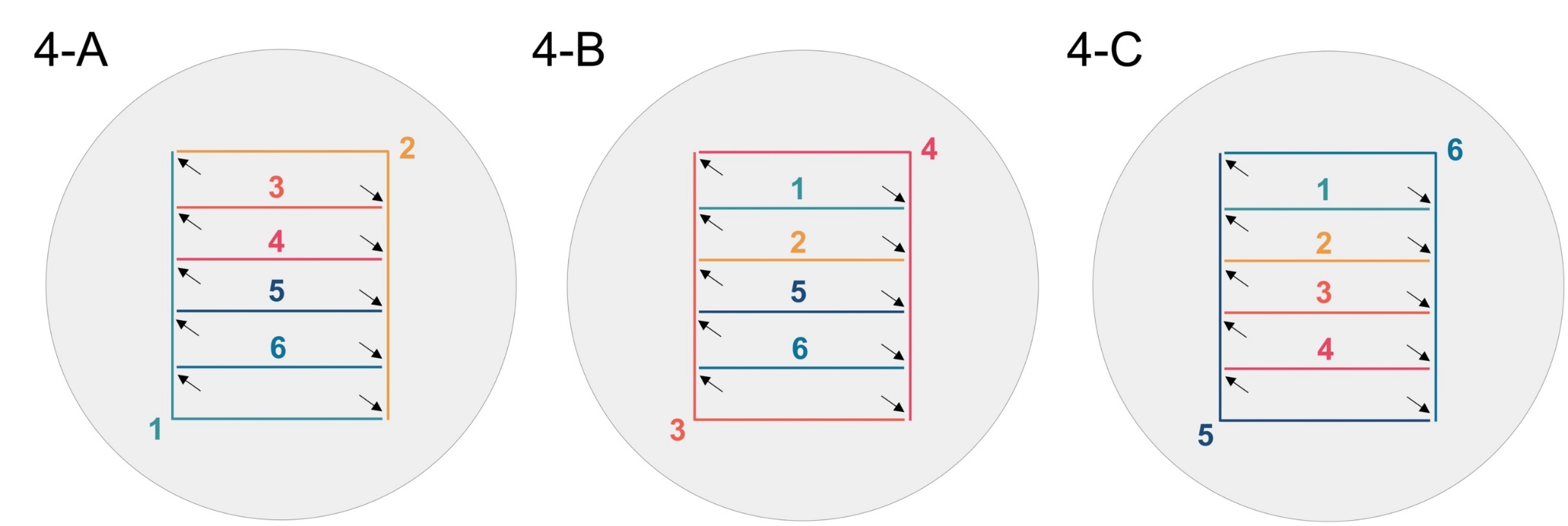


**Supplementary Figure 4:** The cross-streaking assay, an illustration of 3 different patterns: pattern 1 (4-A), pattern 2 (4-B), and pattern 3 (4-C) in experiment and demonstration of 6 streaked lines of LAB. Close contact points are marked as a black arrow.

Note: (1) = *L. acidophilus* KMP, (2) = *L. plantarum* KMP, (3) = *P. pentosaceus* KMP, (4) = *L. plantarum* CU31-5B, (5) = *P. pentosaceus* CU115, and (6) = *E. faecium* CU28-1M
